# Development and evaluation of a technology-enhanced simulation to measure physician decision making in trauma triage

**DOI:** 10.1371/journal.pone.0353381

**Published:** 2026-07-22

**Authors:** Deepika Mohan, Galen Switzer, Baruch Fischhoff, Jonathan Elmer, Kimberly J. Rak, Jacqueline L. Barnes, Douglas B. White

**Affiliations:** 1 Department of Surgery, University of Pittsburgh School of Medicine, Pittsburgh, Pennsylvania, United States of America; 2 Department of Critical Care Medicine, University of Pittsburgh School of Medicine, Pittsburgh, Pennsylvania, United States of America; 3 Department of Medicine, University of Pittsburgh School of Medicine, Pittsburgh, Pennsylvania, United States of America; 4 Department of Engineering and Environmental Policy, Carnegie Mellon University, Pittsburgh, Pennsylvania, United States of America; 5 Department of Emergency Medicine, University of Pittsburgh School of Medicine, Pittsburgh, Pennsylvania, United States of America; 6 Department of Neurology, University of Pittsburgh School of Medicine, Pittsburgh, Pennsylvania, United States of America; Vignan’s Institute of Information Technology, INDIA

## Abstract

**Purpose:**

Variable implementation of clinical practice guidelines causes preventable morbidity and mortality. The paucity of valid and reliable measures of physician performance impedes efforts to improve implementation. The objective of this study was to evaluate a low-cost, scalable simulation method to evaluate physician non-adherence to guidelines, using trauma triage in Emergency Departments (EDs) as an archetypal clinical problem.

**Methods:**

We created an online simulation that mimicked the task environment of a non-trauma center ED. We recruited a sample of ED physicians, asked them to use the simulation, and obtained the electronic health records of injured patients treated by these physicians in the prior 3 years. We used signal detection theory, a behavioral science method, to analyze triage performance. The method quantified the influence of 2 determinants of non-adherence: perceptual sensitivity (diagnostic accuracy) and decisional threshold (revealed preferences for false positive/negative decisions). We collected evidence of the simulation’s response process, internal structure, content validity, and relations with other variables.

**Results:**

Among 180 invited physicians, 60/180 (33%) enrolled and 45/60 (75%) participated; 38/45 (84%) had accessible electronic records. Physicians completed an average of 20/26 simulation trauma cases, spending 2.6 minutes/case (SD 1.8), and making 2.6 decisions/case (SD 1.7). Responses to similar types of simulation cases were consistent (Cronbach’s alpha 0.80–0.86). Users reported strong content validity. Perceptual sensitivity on the simulation and in real-life correlated moderately well among physicians who evaluated ≥10 severely injured patients/year (rho 0.46, 95% CI 0.07–0.76, p = 0.025), but not lower volumes (rho −0.26, 95% CI −0.87–0.50, p = 0.39); decisional thresholds were uncorrelated (rho 0.22, 95% CI −0.12–0.51, p = 0.18).

**Conclusions:**

The technology- and behavioral science-enhanced simulation demonstrated evidence of validity across multiple domains, with mixed findings for relations with other variables. With further refinement, it offers a promising avenue for studying physician decision making in trauma triage, with possible application to other clinical domains.

## Introduction

Clinical practice guidelines reduce inappropriate practice variation, and enhance the efficiency and quality of health care [1]. Physicians may not follow practices recommended in clinical practice guidelines for many reasons, including appropriate clinical judgment, diagnostic errors, situational awareness, risk preferences, and environmental pressures [2,3]. Developing interventions to improve performance requires identifying and addressing potentially malleable determinants of behavior [4,5]. Existing tools to assess decision making, or clinical reasoning, have important limitations [6–8]. For example, non-workplace based assessments, such as licensing exams, have strong evidence of content validity, but do not represent authentic clinical reasoning activities [8]. In contrast, methods like direct observation capture the task well, but have a wide range of inter-rater reliability and limited scalability [9]. The non-systematic nature of clinical practice further complicates the measurement of performance in the real world because low base rates often preclude inferences about the causes of observed behavior [10].

We reasoned that integrating behavioral science analytic methods with advances in simulation might overcome the limitations of existing methods of measuring performance. We selected triage in the Emergency Departments (EDs) of non-trauma centers as an archetypal clinical problem, where better ability to measure and to analyze physician performance could improve patient outcomes. Injury is the leading cause of loss of life among people younger than 45 and a major threat to the independence of those over the age 65 [11,12]. Receiving treatment at Level 1 and 2 trauma centers reduces morbidity and mortality among the severely injured [13,14]. Half of all injured patients present initially to the emergency departments (EDs) of non-trauma centers, where physicians must evaluate them quickly, identify those with severe injuries, and arrange for their transport to a higher level of care [15,16]. Between 30 to 70% of severely injured patients who present initially to a non-trauma center will remain there, under-triaged, in large part because of imperfect physician decision making [17,18].

We combined a technology-enhanced simulation of the clinical environment with signal detection theory, a behavioral science method of quantifying the influence of cognitive processes on behavior, to evaluate the decision making of ED physicians. Here, we report results from a study to collect evidence of the validity of the simulation’s response process, internal structure, content, and relations with other variables, and discuss the inferences permitted by this evidence.

## Methods

### Overview

We evaluated a novel technology-enhanced simulation tool, which we named meaSuring perfOrmance iN trAuma tRiage (SONAR), with a convenience sample of physicians working for a large health system in western Pennsylvania. The University of Pittsburgh’s Human Research Protection Office approved the study (STUDY24020164). Physicians were recruited between 3 September 2024–6 December 2024. All participants provided written informed consent electronically before completing the biographical survey. We used the STARD 2015 reporting guidelines to structure the manuscript.

### Conceptual framework

#### Signal detection theory.

Trauma triage requires physicians to make decisions under uncertainty, balancing the competing risks of under- and over-triage. Conventional approaches to evaluating triage performance typically rely on aggregate measures such as proportions of under- and over-triage [19]. However, these measures conflate two distinct determinants of decision making: the ability to distinguish between severely and minimally injured patients and the propensity to favor transfer versus non-transfer when uncertainty exists. Signal detection theory resolves this problem by allowing the analysis of the latent constructs informing dichotomous decisions made under conditions of uncertainty and time-pressure. The method has improved understanding of diagnostic performance across multiple domains, including threat detection by Transportation Security Administration officers, cancer detection by radiologists, and college students’ recognition of situations posing a risk of sexual violence [20–22]. Application in this context offers novel opportunities for designing effective behavioral interventions in trauma triage [23].

**Fig 1:**
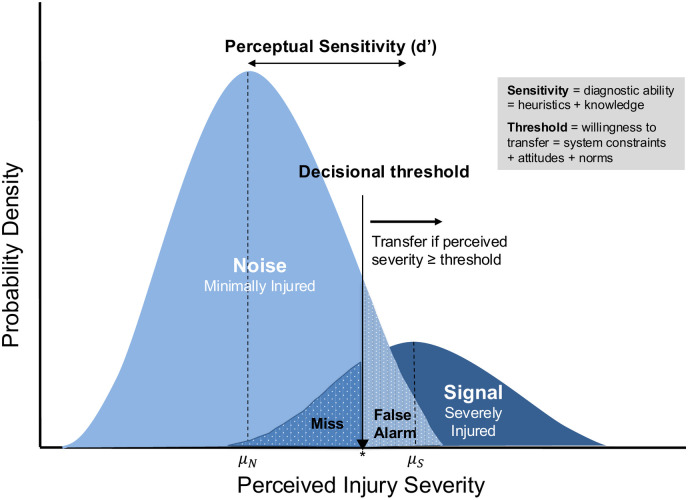
Schematic of signal detection theory. Signal detection theory posits that physicians perceive injuries as falling along a continuum ranging from very minor to severe. The signal distribution shows their perception of severely (signal) and minimally injured (noise) patients. The further apart these distributions, the greater their perceptual sensitivity. The decisional threshold is the point on the continuum above which physicians transfer patients. If perceptual sensitivity remains constant, then as the decisional threshold moves to the right, physicians tend to err towards false negative decisions (not transferring patients with the signal of serious injury).

Signal detection theory decomposes diagnostic decisions into two components: perceptual sensitivity and decisional threshold [24]. Perceptual sensitivity quantifies the ability to distinguish between ‘signal’ and ‘noise’, which in the context of trauma triage represents the ability to distinguish patients with severe or minor injuries. Higher values indicate greater discrimination ability. Decisional threshold quantifies the tendency to err on the side of false negative or false positive decisions, which in this context reflects a willingness to prefer under- or over-triage. Negative values indicate a liberal transfer threshold, or a preference for over-triage; positive values indicate a restrictive transfer threshold, or a preference for under-triage [25]. Thus, signal detection theory separate two constructs, each with different antecedents and representing distinct targets for intervention. Perceptual sensitivity reflects knowledge and heuristics. Decisional thresholds arise from extrinsic constraints and personal attitudes (e.g., risk tolerance, acceptance of clinical practice guidelines) [25]. **Fig 1** depicts the application of signal detection theory to trauma triage.

#### Validity framework.

**Fig 2:**
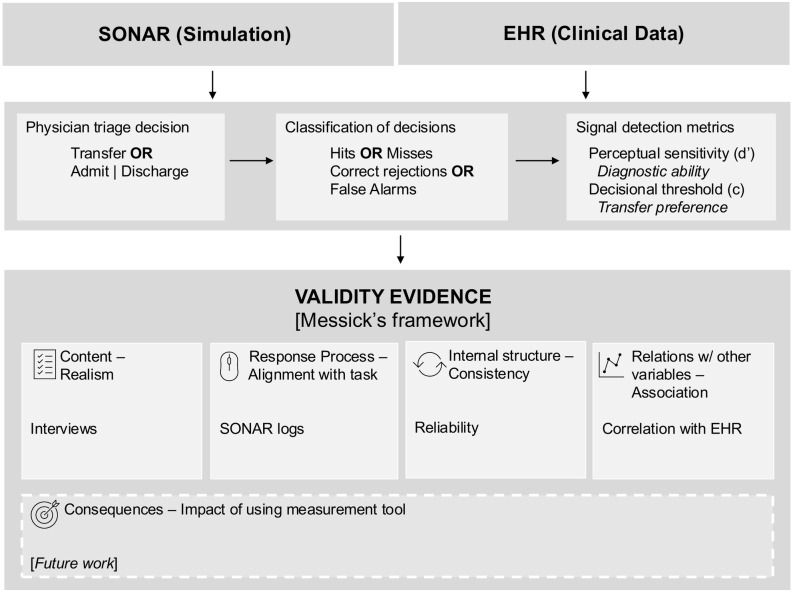
Conceptual framework linking simulation-based assessment to validity evidence. Physicians make triage decisions in both a simulation environment (SONAR) and clinical practice (electronic health record [EHR] data). Decisions are classified as hits, misses, false alarms, and correct rejections, and analyzed using signal detection theory to derive perceptual sensitivity (d′), reflecting diagnostic ability, and decisional threshold (c), reflecting transfer preference. Validity evidence for these measures is evaluated across domains of Messick’s framework, including content, response process, internal structure, and relations with other variables, with consequences identified as an area for future study.

We used the framework established by Messick to evaluate the validity of our simulation, which was adopted by the American Educational Research Association and American Psychological Association as their standard in 1999 and again in 2014 [26]. This framework describes 5 domains of validity evidence: content (a description of the steps to ensure the assessment content reflects the intended construct), response process (how well the user actions align with the intended construct), internal structure (reliability), relation to other variables (association between assessment scores and other measures with which the scores have a specified theoretical relationship), and consequences (impact of the assessment and the decisions that result) [27]. We assessed these domains as follows: content validity through user opinions; response process through analysis of user actions during the simulation; internal structure through reliability analysis of disposition decisions across case types; and relations with other variables through comparison of simulation-derived signal detection metrics with physician performance observed in clinical practice. We deferred the evaluation of consequences evidence, reasoning that we needed to understand the other properties of the tool before we could justify studying the consequences of using SONAR for applied purposes. **Fig 2** shows the conceptual framework of the study.

### Participants

We recruited physicians working in the emergency departments of non-trauma centers in a large health system in western Pennsylvania, by asking clinical supervisors to distribute information about the trial to their faculty. Physicians interested in participating completed a written online consent form, answered a biographical questionnaire, and received instructions on how to access SONAR. We provided an honorarium to those who completed the task, which we increased from $100 to $150 after initial recruitment efforts proved unsuccessful, based on anecdotal responses.

### Study protocol

Participants completed the web-based protocol at their convenience. After logging in to a website that hosted the game, they reviewed a brief tutorial, and then used SONAR. We asked them to make decisions on SONAR as if they were working a regular shift. After they completed the tool, we asked them to complete a brief survey to evaluate the acceptability of SONAR. We subsequently purposively sampled participants with restrictive and liberal decisional thresholds, and invited them to participate in semi-structured interviews to evaluate the acceptability, usability, and content validity of SONAR.

### SONAR

**Fig 3:**
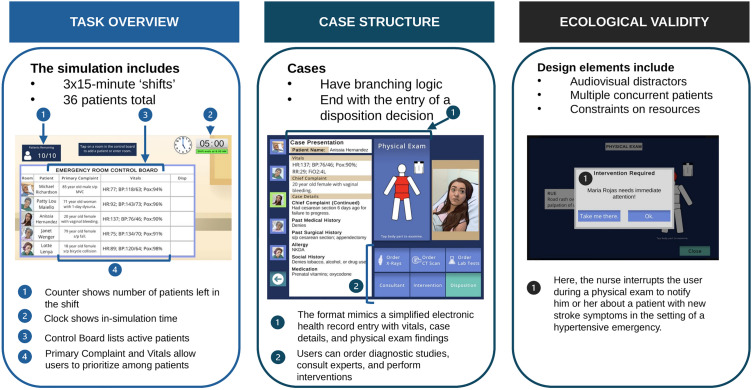
Schematic of SONAR. We show the structure of SONAR, along with screenshots of the different components. Reprinted from the website hosting the simulation under a CC BY license, with permission from the University of Pittsburgh, original copyright 2026.

In previous research, we constructed 140 branching trauma and non-trauma case vignettes based on clinical data, and refined them based on input from a panel of expert trauma surgeons and emergency medicine physicians [28,29]. We selected 60 of these cases, looking for a mix of demographic and clinical characteristics, and collaborated with 1st Playable Productions (Troy, NY) to present them within a 2-D virtual simulation. The simulation (SONAR) presented users with a random sample of 36 of the 60 cases over 45 minutes, such that each user received 13 severely injured cases (mean Injury Severity Score [ISS] 18.5, range 16–29), 13 minimally injured cases (mean ISS 5.6, range 1–11), and 10 non-injury cases as distractors. Each case had a 2-D rendering of the patient, a chief complaint, vital signs that updated every 10 seconds, a case history, and a description of the physical exam. Physicians could request diagnostic studies, select from a set of 25 interventions (e.g., transfuse blood), or consult a specialist. In the absence of appropriate clinical intervention by the user, severely injured and critically ill patients decompensated and died over the course of the ‘shift.’ Each case ended when physicians made a disposition decision, or the patient died. To reduce fatigue during the web-based protocol, we structured the simulation into 3 sequential 15-minute blocks (total duration 45 minutes), with an optional break between blocks. We provide additional details in **S1 File**, and provide a schematic in **Fig 3**.

### Data sources and management

#### Surveys.

Each physician completed a biographical questionnaire at the time of enrollment providing information about their demographics (e.g., sex, race, ethnicity), educational background (e.g., years of experience, board certification, certification in Advanced Trauma Life Support), and practice environment (e.g., number of shifts worked/month). After participants completed SONAR, we asked them to complete a structured assessment of the acceptability of the intervention using the User Engagement Scale–Short Form (a validated 12-item instrument with 5-point Likert scales) [30].

#### SONAR.

We hosted SONAR on a secure server that captured information about user actions during each case, including time spent, diagnostic studies requested, interventions performed, specialists consulted, and disposition decisions. We scored each disposition decision for the trauma cases as adherent or non-adherent with American College of Surgeons clinical practice guidelines, in terms of the signal detection theory metrics [16]. For severely injured patients, we defined adherence as deciding to transfer the patient to a higher level of care (hit) and non-adherence as deciding to discharge or admit the patient (miss). For minimally injured patients, we defined adherence as deciding to discharge or to admit the patient to the local hospital (reject) and non-adherence as deciding to transfer the patient to another hospital (false alarm).

#### Chart review.

An institutional data warehouse provided us with de-identified medical records for patients evaluated at non-trauma centers within the health system from 1 January 2021–31 October 2024, who had at least one ICD10-CM code for injury. They accessed the data for us twice: 26 September 2024 and 19 December 2024. We extracted diagnosis codes, demographics, and discharge disposition status from the discharge abstract. We obtained the names of treating physicians from claims filed for each patient. We restricted our cohort to patients treated by study participants. We translated ICD10-CM codes into ICD9-CM codes, using a generalized equivalency matrix published by the Centers for Medicare and Medicaid, and then ran ICDPIC, a validated algorithm for translating ICD9-CM codes into injury severity scores (ISS) [31]. We selected patients with an ISS ≥ 1, categorizing them as minimally (ISS < 16) or severely (ISS ≥ 16) injured. Finally, we coded the disposition decisions into hits, misses, rejections, and false alarms as for SONAR. We describe data management in more detail in **S1 File**.

#### Post-SONAR debriefing interviews.

We interviewed a subset of our study participants regarding their perceptions of SONAR’s acceptability, usability, and content validity. Our multi-disciplinary team, with expertise in behavioral science, qualitative research methods, instrument development, trauma surgery, and emergency medicine, developed an interview guide to structure the interview (**S1 File**). Two experienced qualitative investigators (KJR, JLB) conducted the interviews, which were recorded and transcribed, and supplemented with hand-written notes. They reviewed preliminary transcripts and the notes in collaboration with DM, ending data collection after 12 interviews when the team felt that it had achieved coding saturation on the main question of acceptability [32]. We de-identified and then coded the transcripts using NVivo 1.6.1 (QSR International, Melbourne, Australia) software for data management.

### Analyses

#### Physician sample.

We summarized physician characteristics using means (standard deviations [SD]) for continuous variables and counts (percentages) for categorical variables. We calculated the response rate as the proportion of physicians who played SONAR, among those who agreed to participate. We included all physicians who initiated SONAR (i.e., partial completers) to maximize use of available data and reduce selection bias; however, analyses of specific outcomes were restricted to physicians with sufficient data to calculate the relevant metrics, as described below. Missing data were not imputed, given the small sample size and the exploratory nature of the study; analyses were therefore conducted using available data. All analyses were conducted in STATA 17 (Statacorp, TX).

#### Evidence of response process and internal structure.

We summarized the number of cases completed, the time spent on each case, and the types of decisions (e.g., diagnostic, disposition) made. We measured the consistency with which physicians made disposition decisions for severely and minimally injured patients (i.e., internal reliability) using Cronbach’s alpha coefficient.

#### Acceptability and content validity.

We summarized responses to the User Engagement Scale–Short Form. During the interviews, to better understand acceptability, we investigated three of the questions on the instrument in more detail: “using SONAR was worthwhile”; “using SONAR was taxing”; “I felt frustrated while using SONAR.” Additionally, we investigated perceptions of the content validity of SONAR, asking them how realistic they found the experience.

#### Evidence of relations with other variables.

We calculated signal detection metrics for each physician in the real world and on SONAR with the following equations [25].


$$\begin{array}{c} Perceptual\;sensitivity\;(d^{\prime })\;=ln(hit\;rate)\;-\;ln(miss\;rate)\;-\;ln(false\;alarm\;rate)\;+\;ln\;(reject\;rate) \end{array}$$ (1)

$$\begin{array}{c} Decisional\;threshold\;(c)\;=-ln(false\;alarm\;rate)\;+\;ln(reject\;rate) \end{array}$$ (2)

Briefly, as described above, perceptual sensitivity (d’) reflects the ability to discriminate between severely and minimally injured patients, whereas decisional threshold (c) reflects the clinician’s tendency to transfer versus not transfer patients independent of discrimination. We corrected for perfect scores by converting proportions of 0 to $\frac{\text{1}}{\text{2}}$N and 1 to 1- $\frac{\text{1}}{\text{2}}$N, where N equaled the total number of cases (or patients) each clinician had evaluated.

We compared the individual-level correlation between the metrics in the real world and on SONAR using the Spearman correlation coefficient, selected a priori given the small sample size and potential non-normal distribution of the metrics. We report correlation coefficients with 95% confidence intervals (CIs) to interpret the precision of the estimates, generated using bootstrap resampling (1000 iterations). In a planned sensitivity analysis, we repeated the analysis for physicians who evaluated ≥10 severely injured patients/year (high-volume) or <10 severely injured patients/year (low volume).

We designed the study to capture a moderate correlation (rho = 0.4–0.6) between signal detection theory metrics measured using SONAR and in the real world with an alpha of 0.05 and a power of 80%, using Cohen’s power primer for behavioral studies. We, therefore, estimated that we would need to recruit 64 participants [33].

## Results

### Physician sample

Among the 180 physicians invited to participate, 60/180 (33%) completed the consent form, 45/60 (75%) of whom used SONAR. Physicians had a median of 13 years of experience (IQR 6–37). Most (82%) were board-certified in emergency medicine; all (100%) had completed ATLS; and most (75%) worked ≥11 shifts per month. We subsequently interviewed 12 physicians, recruited based on their decisional thresholds on SONAR. Additional physician characteristics are reported in **Table 1**.

**Table 1:** Physician characteristics.

Characteristics		Sample N = 60
Gender, n (%)		
Female		17 (28)
Male		42 (70)
Prefer not to say		1 (2)
Race, n (%)		
Asian		6 (10)
Black		4 (7)
White		45 (75)
Prefer not to say		5 (8)
Ethnicity, n (%)		
Hispanic		3 (5)
Prefer not to say		3 (5)
Residency, n (%)		
Emergency Medicine		49 (82)
Family Practice		6 (10)
Internal Medicine		4 (7)
Other		1 (2)
Experience, median (IQR)		13 (6-37)
Have you completed ATLS – yes, n (%)		60 (100)
When did you complete ATLS, n (%)		
<1 year ago		4 (7)
1-4 years ago		20 (33)
>4 years ago		36 (60)
Number of shifts worked per month, n (%)		
5-10		15 (25)
11+		45 (75)

### Evidence of SONAR’s validity

#### Response process and internal structure.

Physicians completed an average of 20 of their 26 trauma cases (SD 10.3) during the 45 minute session, spending an average of 2.6 minutes (SD 1.8) reading and responding to each case. They encountered a mean of 1.5 distractions (SD 2.5) per case and made a mean of 2.8 decisions (SD = 1.7), requesting diagnostic tests for 82% of cases, performing interventions for 65% of cases, and consulting specialists for 22% of cases. They transferred 84% of severely injured patients and 37% of minimally injured ones. On SONAR, physicians had a median perceptual sensitivity of 1.9 (IQR 1.23–2.51), indicating good ability to distinguish between minimally and severely injured patients. They had a decisional threshold of 0.47 (IQR 0.15–1.20), indicating a tendency over-triage.

Physicians made similar disposition decisions for cases that described minimally and severely injured patients, suggesting the instrument had strong internal reliability (Cronbach’s alpha 0.80 [minor] and 0.86 [severe]).

#### Acceptability and Content Validity.

Among physicians who used SONAR, 36 (80%) completed the User Engagement Scale. See **Table 2** for the full responses. Most (≥80%) found it interesting, worthwhile, and absorbing. About 30% of the respondents described finding it taxing and 56% described the task as frustrating.

**Table 2:** Responses to the user scale engagement questionnaire (n = 36).

Item^1^	Proportion who agreed or strongly agreed, n (%)
I felt interested in this experience	32 (89)
My experience was rewarding	27 (75)
Using *SONAR* was worthwhile	31 (86)
*SONAR* appealed to my senses	25 (69)
*SONAR* was aesthetically appealing	32 (89)
*SONAR* was attractive	22 (71)
Using *SONAR* was taxing	12 (33)
I found *SONAR* confusing to use	13 (36)
I felt frustrated while using *SONAR*	20 (56)
I was absorbed in the experience	32 (89)
The time slipped away	28 (78)
I lost myself in the experience	22 (61)
Summary score^2^	median 3.75 (IQR 3.4–3.8)

^1^Respondents selected from a Likert scale: 1 = strongly disagreed; 2 = disagreed; 3 = neutral; 4 = agreed; 5 = strongly agreed.

^2^Highlighted items were reverse coded when included in the summary score (e.g., strongly agreed [5] was translated into strongly disagreed [1]).

In the semi-structured interviews, almost all respondents described SONAR as worthwhile for reasons that ranged from “I am fascinated with being efficient in the emergency department” [Subject 03] to “it made me think of how I am approaching these trauma patients in my ED and if there are patients that we under-triage” [Subject 08]. The one interview subject who did not find SONAR useful described it as “too artificial” [Subject 09].

People who found the task taxing described a variety of causes including the time-pressure, the need to track multiple patients simultaneously, and the distractors. However, they did not necessarily consider that a disadvantage: “It was taxing. I mean, honestly, I can tell you it was very, very taxing. I mean, not in a bad way, but it used a lot of mental capacity to do it...I don’t know how many minutes it was, 15, 20, whatever, but the whole time I was using brain power to put everything together” [Subject 01]. Physicians who reported frustration usually described struggling to navigate the user interface and “learning how to play the game while trying to make decisions at the same time” [Subject 04].

Almost all interview participants described the simulation as having strong content validity: “I liked the SONAR simulation in that it did a really good job of recreating the chaos” [Subject 11]. **Table 3** provides additional qualitative impressions of SONAR.

**Table 3:** Qualitative impressions of SONAR.

How similar were game decisions to real life	It’s very similar. Yeah. The game isn’t real life, so real life is different. But the thinking is the same in real life, in the game. And the game allows you to put it in hyper-speed decision making. [Subject 03]
How taxing did you find the simulation	I found it taxing, and I felt like the amount of taxing was pretty close to what the real life experience is. The time obviously moved a lot faster, which kind of gives you that sense of urgency, you know. [Subject 11]
How frustrating did you find the simulation?	Can you elaborate on what you mean by frustration? So, if you mean like you have all of these patients all at the same time and all of them need your attention and you’re trying to figure out how to prioritize and then that interrupter thing pops up. Then you’re like trying to simulate like the real level of frustration that I feel in my job, then very accurate. If you mean like there was some kind of problem with the system, I didn’t really experience that. [Subject 11]
How worthwhile did you find the simulation?	I think it was a really good experience. It definitely made me think of how I am approaching these trauma patients in my ED and if there are patients that we are under triaging. Or not considering like higher level of care just because they’re not like super unstable. So, I think it was good for me to reflect on my clinical practice. [Subject 08]

#### Relations with other variables.

Among the 45 trial participants, 38 worked at non-trauma centers that submitted electronic health records to the institutional data warehouse. They evaluated a median of 588 injured patients (inter-quartile range [IQR] 319−826) per year during the study period. They saw a median of 9 patients (IQR 7−18) with severe injuries and transferred a median of 2 (IQR 1−4). They saw a median of 579 patients (IQR 315−816) with minor injuries and transferred 15 (IQR 9−31). In practice, they had a perceptual sensitivity of 2.44 (IQR 1.9–2.72), indicating a good ability to distinguish between severely and minimally injured patients, and a decisional threshold of 3.38 (IQR 3.04–3.8), indicating a reluctance to transfer patients to a higher level of care.

**Fig 4:**
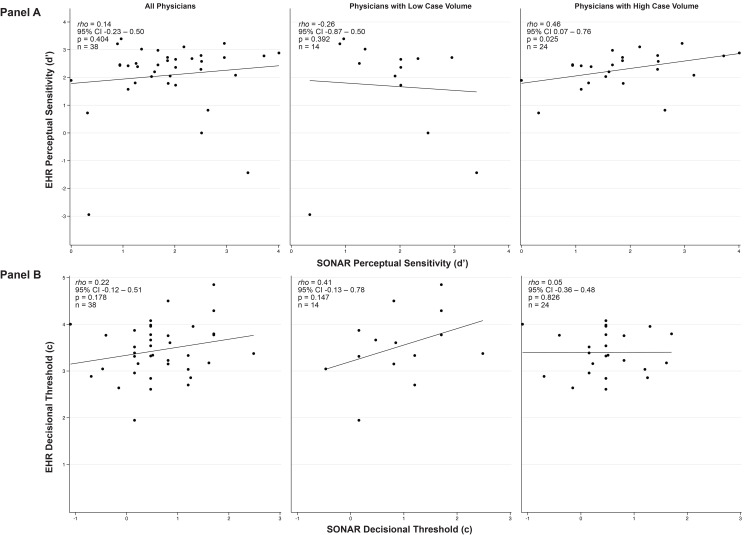
Comparison of physicians’ signal detection theory metrics measured on SONAR and using electronic health record data. In Panel A, we show perceptual sensitivity for the whole cohort, along with the subsets of those with low and high case volume. In Panel B, we show the decisional thresholds.

Among physicians with electronic health record data, there was no correlation between perceptual sensitivity in practice and on SONAR. However, in the prespecified sensitivity analysis, among physicians who treated ≥10 severely injured patients a year, there was a moderate correlation (rho = 0.46, 95% CI 0.07–0.76, p = 0.025). Among physicians who treated fewer patients, there was no correlation (rho = −0.26; 95% CI −0.87–0.50, p = 0.39). Decisional thresholds on SONAR and in the clinical record were uncorrelated, both in the whole sample and the high or low clinical volume subsamples. **Fig 4** shows the correlations.

## Discussion

We attempted to overcome limitations in existing measures of physician performance by integrating behavioral science analytic methods with technology-enhanced simulation, using the clinical problem of trauma triage as a test case. In pre-specified analyses aligned with Messick’s framework, we found evidence of validity in three of the four domains: response process, internal structure, and content. In the fourth domain, relation to other variables, we found mixed evidence of validity. Specifically, perceptual sensitivity (i.e., diagnostic performance) on the simulation correlated with real-world performance among physicians with high caseloads but not among those with caseloads, and decisional thresholds (i.e., willingness to transfer) did not correlate with real-world behavior. Taken together, these findings provide preliminary, confirmatory evidence that the simulation has sufficient evidence of validity for the limited purpose of informing the development and testing of behavioral interventions designed to address diagnostic performance, while not yet supporting its use as a surrogate for workplace-based assessment.

### Possible implications

The simulation offers a potential advance over existing methods of physician assessment by enabling measurement of the causes of non-adherence with clinical practice guidelines. Most simulations assess technical skill [6]. The few that address clinical reasoning typically focus on accuracy as their primary metric of performance [8]. However, decisions to deviate from clinical practice guidelines can have multiple causes; behavioral interventions that target the wrong cause can produce unintended, potentially adverse consequences [34–36]. Our application of signal detection theory allows us to overcome this limitation by deconstructing decision making into two components, each informed by different antecedents and responsive to different types of interventions. The simulation therefore has the potential to promote a theory-based approach to reducing non-adherence to clinical practice guidelines, consistent with best-practice recommendations for the development of behavioral interventions [37].

An additional strength of SONAR is the presence of at least mixed evidence of relations with separate measures. In comparison, a systematic review of studies evaluating the validity of technology-enhanced simulations found that less than 1/3 of them attempted to meet the standard of establishing evidence of validity [6]. The few with evidence of relations with separate measures reported correlations similar in size (i.e., moderate) to those that we found [38–40].

### Explanations of findings

We speculate that the lack of correlation in perceptual sensitivity metrics among physicians with low caseload reflects the study’s failure to achieve its target sample size (64 planned versus 45 enrolled, with 38 included in key analyses), which likely reduced statistical power. However, alternative explanations include the lack of precision inherent in the estimation of real world performance among physicians with low case volume [10], and the potential failure of the simulation to capture differences in their decision making. The latter raises concerns about the generalizability of the simulation to all physicians. These hypotheses warrant further investigation

Similarly, the absence of correlation between decisional thresholds on the simulation and in real life may also reflect multiple factors. The limited sample size may have introduced instability in the estimates. Alternatively, aspects of the simulation design may also have caused a measurement mismatch between the simulation and real-world data. Most applications of signal detection theory in medicine have focused on problems of visual perception, such as breast cancer diagnosis, because the method requires large numbers of observations [23,25]. When using signal detection theory to understand clinically complex decisions, therefore, researchers must balance task validity with respondent burden. We have experience building technology-enhanced simulations to study trauma triage, and in the past attempted to maximize verisimilitude, analyzing performance using more standard metrics such as accuracy [28]. For SONAR, however, we simplified clinical details to encourage faster responses. For example, instead of allowing users to select from among 250 possible diagnostic and therapeutic options to manage a patient as in previous builds, we provided 25. This allowed physicians to respond more rapidly than when the task environment better reflected the real world. However, eliminating the friction involved in making disposition decisions may have introduced to measurement error. Other researchers should consider this trade-off carefully, determining how best to balance experimental efficiency with ecological validity for their specific clinical problem.

### Limitations of study

This study had several limitations. First, we recruited a local convenience sample from a highly inclusive trauma system to establish validity evidence [41]. We made the decision pragmatically because access to electronic health records for this cohort facilitated the collection of evidence of relations with other variables. However, this approach may limit generalizability among physicians practicing in different systems or resource environments. Second, only one-third of eligible physicians agreed to participate in the trial, and only 75% of those who agreed to participate completed the task, potentially introducing selection bias. Participants may have systematically differed from non-participants in ways that could have influenced both estimates of validity evidence as well as observations about the types of cognitive processes used to make trauma triage decisions. Anecdotally, we learned that contractions in the health system’s work force may have influenced physicians’ willingness to assume non-essential professional obligations. We therefore have no reason to believe that non-responsiveness occurred systematically, but cannot discount the possibility. Third, we interviewed only a subset of participants, stopping when we had reached thematic saturation on the main question of acceptability, as specified by best-practices in qualitative research [42]. However, the small numbers meant we could not investigate causes of the discordance between measured and observed decisional thresholds. Finally, we did not evaluate the consequences of using SONAR, an important domain within Messick’s framework. Next steps will include assessment of the simulation as a means of informing, personalizing and testing behavioral interventions in trauma triage, as well as the consequences (e.g., resource utilization) of using the simulation for this purpose.

## Conclusions

We found preliminary confirmatory evidence supporting the validity of a technology- and behavioral science–enhanced simulation to measure physician performance in trauma triage. However, mixed evidence for relations with other variables underscore the need for cautious interpretation of results and further validation efforts. With further refinement and evaluation, the simulation offers a promising avenue for non-workplace-based assessment in trauma triage, for informing the design of behavioral interventions in trauma triage, and for generalizability to other clinical domains.

## Supporting information

S1 File Additional methods. The file includes details about the methods used to develop SONAR, to interpret output, to clean electronic health record data. It also includes the interview guide used to debrief study participants about the acceptability of the simulation.
(DOCX)
