# Supplementary material for: Development and evaluation of a technology-enhanced simulation to measure physician decision making in trauma triage
Source: PLoS One. 2026 Jul 22;21(7):e0353381. doi: 10.1371/journal.pone.0353381 (PMC13390866; doi:10.1371/journal.pone.0353381)
Supplement: S1 File — The file includes details about the methods used to develop SONAR, to interpret output, to clean electronic health record data. It also includes the interview guide used to debrief study participants about the acceptability of the simulation. (DOCX) [file pone.0353381.s001.docx]

**Supplementary File 1 – Development and evaluation of a technology-enhanced simulation to measure physician decision making in trauma triage**

[SONAR (simulation) 3](#_Toc227746457)

[Configuration 3](#_Toc227746458)

[Operational Parameters 4](#_Toc227746459)

[Design Decisions 4](#_Toc227746460)

[Data Management 5](#_Toc227746461)

[Electronic Health Record (clinical) data 6](#_Toc227746462)

[Overview 6](#_Toc227746463)

[Data Management 6](#_Toc227746464)

[**Step 1. Generating injury severity scores (ISS)** 6](#_Toc227746465)

[**Step 2. Finding patients treated by trial physicians** 7](#_Toc227746466)

[**Step 3. Categorizing the transfer status of each patient** 7](#_Toc227746467)

[**Step 4. Estimating signal detection metrics** 7](#_Toc227746468)

[Interview Guide 9](#_Toc227746469)

[REFERENCES 11](#_Toc227746470)

# **SONAR (simulation)**

## **Configuration**

SONAR is a 2-D simulation designed in collaboration with 1st Playable Productions (Troy, NY). We provided the game development company with a set of 140 branching clinical cases developed based on clinical data and then refined across multiple prior projects to ensure accuracy [1-3]. We labelled each case as trauma or non-trauma, and then for trauma cases with the injury severity score. Each case included the patient's state (e.g., hemodynamically stable), clinical details (e.g., demographics, history and physical exam, injury severity score), radiographic findings if relevant (e.g., x-rays, CT scans, MRI studies), laboratory data, and state changes with and without interventions. We provided the company with data for normal radiographic and laboratory values. If users requested these studies on a patient without an abnormal finding, they would receive the normal laboratory values or report. To improve verisimilitude, the simulation would return a laboratory value selected from within the normal range. We also provided data on parameters for different physiologic states, and responses to the 25 interventions that we made available to users (e.g., insertion of chest tube, administration of blood, administration of vasopressors).

We selected 60 of the 140 cases for development during this project, balancing demographic and injury characteristics. The company transformed the case data into a simulacrum of an electronic health record, along with a picture of the patient, and embedded the records within the simulation. The simulation opened with instructions, followed by a tutorial and then an opportunity to experiment with the user interface. The user then reviewed the Emergency Room Control Board, which listed 5 cases that the user had to evaluate and treat. The Board listed a primary complaint, plus vital signs. By clicking on a patient, the user would jump into a specific room. When in that room, the user could review electronic health record, could order laboratory or radiology studies, consult a specialist, perform an intervention, or make a decision about the disposition of the patient. Vital signs in the record updated every 15 seconds. Physical exam data appeared within a paper doll with highlighted areas denoting abnormal findings. To see a different patient, the user could return to the Control Board or jump to another room listed on the left margin. Cases ended when the user made a disposition decision (e.g., admit, discharge, transfer) or when the patient died.

## **Operational Parameters**

We hosted the simulation on a secure server, which users could access by logging into the study website. We asked users to complete the simulation at their convenience, but ideally when they could spend at least 45 minutes on the task. To reduce fatigue during the web-based protocol, we structured the simulation into 3 15-minutes blocks. Users had the option of taking a break between blocks but not within a block. We asked users to complete the simulation as if they were working at their primary place of employment.

## **Design Decisions**

We included several design elements to increase the simulation’s verisimilitude. At the start, users could select the consultants they would ordinarily have available at their hospital. We programmed consultants to ask the user for their disposition decision before providing any advice and to provide a contradictory opinion in half the cases. Patients arrived at pre-specified but unpredictable intervals, so that physicians had to manage multiple patients concurrently. Physicians could select the order in which they saw patients, based on their physiologic status as represented on a tracking board. New information appeared with a time delay, intended to replicate actual experience, scaled to the simulation. A clock at the top of the screen allowed players to track the passage of time. Physicians could not make a disposition decision for hemodynamically unstable patients, to prevent them from deflecting responsibility for such cases. In addition to their clinical responsibilities, players had to respond to audio-visual distractors that ranged from the alarms of medication pumps to paging alerts to nurses who asking for help with patients in the waiting room. These distractors appeared at a rate of 1 per minute.

## **Data Management**

We hosted SONAR on a secure server hosted by the University of Pittsburgh. User actions uploaded to a database capturing cases presented, cases reviewed, time spent on each case, orders placed, consultant feedback received, distractions encountered, tests reviewed, and the disposition status of each case. For cases where the user requested a consultant opinion, the interface asked the user to state their opinion on the disposition decision before the consultant responded. The system captured both the original opinion (used for analysis) and the final outcome.

We categorized cases as non-trauma, minor trauma, and severe trauma based on labels attached to each case in the simulation. We dichotomized disposition decisions into two categories: transfer = 0 or 1. We categorized outcomes using signal detection nomenclature. We designated severely injured patients as a 'hit' if transferred or a 'miss' if not transferred. We designated minimally injured patients as a 'correct rejection' if not transferred' or a 'false alarm' if transferred. We collapsed cases at the level of the study provider, summarizing the total, severely and minimally injured patients that they evaluated along with their outcomes. We transformed the outcomes into proportions that we named as rates (e.g., hit rate = hit/severely injured), and corrected for perfect values (i.e., 0 or 1) by adding or subtracting $1\div(2*total injured patients)$. Finally, we calculated d' (perceptual sensitivity) and c (decisional threshold) for each provider on SONAR using the following equations [4]:

| $Perceptual sensitivity (d') =$ | $ln(hit rate) - ln(miss rate) - ln(false alarm rate) + ln (reject rate)$ | (1) |
| --- | --- | --- |
| $Decisional threshold (c) =$ | $-ln(false alarm rate) + ln(reject rate)$ | (2) |

# **Electronic Health Record (clinical) data**

## **Overview**

We used UPMC electronic health records to measure physicians' triage performance in the real world. We asked the University of Pittsburgh's Clinical Trial Office – Health Record Research Request (R3) service to search their clinical data warehouse for patients, 18 years or older, who presented to the ED of any UPMC facility between 1 January 2021 to 31 October 2024, with an ICD10 discharge code between S00 to T88 (i.e., an injury diagnosis). The service provided us data on each patient's hospital encounter in 5 text-delimited files: [1] diagnosis codes for the patient's encounter; [2] physicians listed as the attending of record; [3] physicians who filed claims during the encounter; [4] the discharge abstract with dates of admission, discharge, patient class (e.g., inpatient), admission type (e.g., emergency), facility, chief complaint, insurance status, discharge disposition (e.g., home), and discharge destination (e.g., name of nursing home, name of hospital); [5] demographics (age, sex, race) and vital status (date of death); We imported the files into STATA 17.0 (Statacorp, TX), and cleaned them as follows.

## **Data Management**

### **Step 1. Generating injury severity scores (ISS)**

We restricted the cohort to the records with an ICD10 diagnostic code that indicated initial evaluation for an injury (i.e., "S" code with a suffix restricted to "A" or "B"). We removed duplicated records, and used STATA's ICD program to clean the format of the diagnosis codes. We mapped the ICD10 to ICD9 codes using a matrix provided by the Centers for Medicare and Medicaid, and transformed the codes into abbreviated injury scores (AIS) using the publicly available algorithm "icdpic." ICDPIC is an open-access program published by researchers at Maine Medical Center, and subsequently validated independently against nurse registrar-calculated ISS within a regional trauma registry and the Trauma Quality Improvement Program [5-7]. Agreement between the gold standard of nurse registrar estimates and those generated by the program ranges from moderate to substantial (kappa 0.66–0.92), depending on the comparator and the type of trauma (e.g., abdominal versus head). We calculated an ISS for each patient, as the sum of the square of the top three AIS [8]. We categorized patients as having a severe (ISS≥16) or minor (ISS<16) injury, consistent with the literature [9].

### **Step 2. Finding patients treated by trial physicians**

We created a list of the first and last name of providers enrolled in our study, and searched each provider file for their names. We flagged all encounters involving one of our study providers, deleted all encounters without a flag, appended the files, and removed duplicated encounters. This then became our file of study patients.

### **Step 3. Categorizing the transfer status of each patient**

We searched the file of discharge abstracts for study patients, and merged in our estimated ISS values along with demographic data. We used the facility variable to exclude all encounters that occurred at Level 1 or 2 trauma centers within the UPMC network. We created a variable to categorize the transfer status of the patient using both the discharge disposition of the patient as well as the discharge destination. We categorized patients as transferred if the disposition was "Transfer to Hospital" or if the discharge destination was a second hospital.

### **Step 4. Estimating signal detection metrics**

As described for SONAR, we categorized outcomes using signal detection nomenclature. We collapsed the file at the level of the study provider, summarizing the total, severely and minimally injured patients that they evaluated along with their outcomes. We transformed the outcomes into rates, corrected for perfect values, and signal detection metrics using the equations described for SONAR.

# **Interview Guide**

My name is _____and I am a researcher from the department of Critical Care Medicine at the University of Pittsburgh. This study is being conducted by Principal Investigator, Dr. Deepika Mohan, Associate Professor of Critical Care Medicine and Surgery, and colleagues to better understand the environment in which you make triage decisions.

I will be asking you several questions about your experience making decisions about the management of trauma patients in your ED. There are no right answers to these questions. Your responses will be completely confidential and will in no way affect your employment. Although participating in this interview has no direct benefits to you, your responses may help to improve future educational experiences.

We can stop the interview at any point if you feel uncomfortable or do not wish to continue. Also, you do not have to answer any questions that make you feel uncomfortable.

**Do you have any questions at this time? Do I have your permission to audio record our conversation?**

**[Begin recording. State the date, time, and participant identification number]**

**First, I would like to learn your typical approach to deciding where trauma patients should go.**

1. What percentage of your patients are trauma patients [where we define trauma as any kind of physical injury, whether a fall or gunshot wound]?
   1. Of trauma patients, what percentage do you transfer to a higher level of care?
      1. What influences that decision?
   2. What kind of patients would you *always* transfer? [*range of patient characteristics, existing conditions, injury types*]
   3. What kinds of patients would you *always* admit or discharge?
2. Some patients fall into a gray area where they may or may not be transferred, depending upon circumstances. Can you describe for me what patients that you think would fall into a gray area?
   1. What things would convince you to keep a grey area patient?
   2. What things would convince you to transfer a grey area patient?
3. How likely would you be to transfer a gray area patient?
   1. (Likert scale ...1=not at all likely 2=somewhat unlikely, 3=neutral, 4= somewhat likely, 5 = very likely)
   2. Could you tell me more about that?
   3. Compared to other physicians in your ED, where do you think your threshold for transferring grey area patients falls?
      1. (...1=much less likely to transfer, 2=somewhat less likely to transfer, 3=about as likely to transfer 4= somewhat more likely to transfer 5=much more likely to transfer)

**I’d like to wrap up by asking you about your experience with the SONAR game.**

1. When playing the game, how similar did you feel your game decisions were to how you would act in real life?
   1. What contributed to any potential differences?
2. How taxing did you find the simulation?
   1. What did you find taxing?
3. How frustrating did you find the simulation?
   1. What did you find frustrating?
4. How worthwhile did you find the experience? Tell me more about that.

# **REFERENCES**

1. Mohan, D., et al., *Validating a vignette-based instrument to study physician decision making in trauma triage.* Med Decis Making, 2014. **34**(2): p. 242-52.

2. Mohan, D., et al., *Assessing the validity of using serious game technology to analyze physician decision making.* PLoS One, 2014. **9**(8): p. e105445.

3. Mohan, D., et al., *Serious games may improve physician heuristics in trauma triage.* Proc Natl Acad Sci U S A, 2018. **115**(37): p. 9204-9209.

4. Macmillan, N.A. and C.D. Creelman, *Detection Theory: A User's Guide*. 1991: Cambridge University Press.

5. Greene, N.H., M.A. Kernic, M.S. Vavilala, and F.P. Rivara, *Validation of ICDPIC software injury severity scores using a large regional trauma registry.* Inj Prev, 2015. **21**(5): p. 325-30.

6. Wan, V., et al., *How does Injury Severity Score derived from International Classification of Diseases Programs for Injury Categorization using International Classification of Diseases, Tenth Revision, Clinical Modification codes perform compared with Injury Severity Score derived from Trauma Quality Improvement Program?* J Trauma Acute Care Surg, 2023. **94**(1): p. 141-147.

7. Clark, D.E., A.W. Black, D.H. Skavdahl, and L.D. Hallagan, *Open-access programs for injury categorization using ICD-9 or ICD-10.* Inj Epidemiol, 2018. **5**(1): p. 11.

8. Baker, S.P., B. O'Neill, W. Haddon, Jr., and W.B. Long, *The injury severity score: a method for describing patients with multiple injuries and evaluating emergency care.* J Trauma, 1974. **14**(3): p. 187-96.

9. MacKenzie, E.J., et al., *A national evaluation of the effect of trauma-center care on mortality.* N Engl J Med, 2006. **354**(4): p. 366-78.
